# Breastfeeding Experiences in Australian Mothers of Multiple Birth Infants

**DOI:** 10.3390/nu17101669

**Published:** 2025-05-14

**Authors:** Muja A. Gama, Jacki L. McEachran, Ashleigh H. Warden, Demelza J. Ireland, Donna T. Geddes, Sharon L. Perrella, Zoya Gridneva

**Affiliations:** 1 School of Molecular Sciences, The University of Western Australia, Crawley, WA 6009, Australia; gamamuja@gmail.com (M.A.G.); jacki.mceachran@uwa.edu.au (J.L.M.); ashleigh.warden@uwa.edu.au (A.H.W.); donna.geddes@uwa.edu.au (D.T.G.); sharon.perrella@uwa.edu.au (S.L.P.); 2 ABREAST Network, Perth, WA 6000, Australia; 3 UWA Centre for Human Lactation Research and Translation, Crawley, WA 6009, Australia; 4 School of Biomedical Sciences, The University of Western Australia, Crawley, WA 6009, Australia; demelza.ireland@uwa.edu.au

**Keywords:** multiple birth infants, twins, triplets, breastfeeding, lactation, breastfeeding characteristics, barriers and facilitators

## Abstract

**Background/Objectives**: Breastfeeding multiple birth infants (MBIs) poses unique challenges that require tailored support; however, research on these mothers’ experiences is limited. This study explored the breastfeeding journeys of Australian mothers of MBIs, highlighting barriers, facilitators, and support needs. **Methods**: Data were collected via an online survey (May–August 2024) and included quantitative data on breastfeeding initiation, duration, and challenges, as well as qualitative insights into mothers’ experiences. Thematic analysis was used to identify key themes, and statistical analyses were used to compare breastfeeding outcomes by parity. **Results**: While most mothers (87%) had an antenatal intention to breastfeed, they faced barriers such as latching difficulties (56%), inadequate milk supply (49%), and sore nipples (47%). Preterm births (58%) and neonatal unit admissions delayed the breastfeeding initiation. Most mothers (99%) used electric breast pumps to boost milk supply (68%) and enable expressed breast milk feeding by other caregivers (65% of mothers). While 72% were satisfied with hospital breastfeeding support and some mothers received excellent hands-on support, others felt neglected due to busy staff or conflicting advice. Mothers frequently reported that breastfeeding guidance was geared toward singletons, leaving them unprepared for the challenges of feeding multiples. Mothers’ suggestions for improving care included specialised guidance, better access to lactation support, and in-home practical support to alleviate the burden of feeding and expressing. Additionally, mothers reported that healthcare professionals should be trained to offer practical, non-judgemental support to help mothers navigate the elaborate challenges of breastfeeding MBIs. **Conclusions**: This study underscores the need for early postpartum support and tailored guidelines to enhance MBI breastfeeding outcomes and maternal-infant well-being.

## 1. Introduction

The global incidence of multiple births has increased by one-third since the 1980s, largely due to medically assisted reproduction and delayed motherhood [1]. In Australia, from 2011 to 2022, multiple birth rates remained steady at 2–3%, with 1 in 70 births resulting in multiples, of which 98.8% were twins and the remaining 1.2% were triplets or higher-order multiples in 2022 [2,3]. Multiple pregnancies are considered high-risk, with 63% of twins and 100% of higher-order multiples being born preterm, elevating neonatal health risks [4].

While the World Health Organization (WHO) recommends exclusive breastfeeding for the first six months, followed by continued breastfeeding with complementary foods for up to two years and beyond [5], breastfeeding rates for MBIs are consistently lower than those for singletons across various regions, such as South Korea [6], Japan [7,8], Spain [9], Sweden [10], and Ghana [11]. Published exclusive breastfeeding rates for MBIs at six months range from 4.1% to 21.5%, which is well below the WHO’s target of 50% [5,12]. While breastfeeding is widely recommended for its numerous health benefits, mothers of MBIs face significant challenges like pregnancy and birth complications and inadequate milk supply. MBI families require tailored support and guidelines to address the physical and emotional demands of caring for multiples to enhance breastfeeding outcomes [13,14].

Mothers of MBIs are less likely to initiate breastfeeding or exclusively breastfeed than mothers of singletons [8,15,16]. Theoretically, it may be physiologically possible for most mothers to produce an adequate milk supply for MBIs due to the ‘supply and demand’ mechanism, where increased milk removal stimulates increased milk production [17,18]. However, mothers of MBIs frequently report perceived insufficient milk supply as a barrier to breastfeeding, leading to early formula supplementation or cessation of breastfeeding [1,19,20]. Further, a multiple pregnancy has a higher risk of complications, with preterm birth being the most common [4,21]. Preterm infants often take time to establish breastfeeding due to respiratory difficulties, underdeveloped muscles, immature sucking reflexes, and difficulties with breathing, sucking, and swallowing coordination [22]. Additionally, separation due to the need for neonatal unit admission disrupts early skin-to-skin contact and breastfeeding initiation [23]. Gestational diabetes mellitus, which is more prevalent in multiple pregnancies, causes impaired insulin sensitivity and is associated with delayed secretory activation, reduced milk production, and shorter breastfeeding duration [24,25]. Furthermore, MBIs are typically born via caesarean section (CS), leading to reduced mobility and difficulties in simultaneously caring for multiple newborns and achieving a comfortable position for breastfeeding [23].

Frequent breastfeeding and/or pumping sessions are necessary for sufficient milk production; however, the physical and time demands of breastfeeding and/or pumping breast milk for MBIs can result in maternal stress and exhaustion. For preterm infants, inefficient breastfeeding necessitates supplementary bottle feeding and continued pumping. The logistics of feeding an infant on each breast simultaneously (tandem feeding) often require physical assistance from others and can be overwhelming [1]. Thus, mothers of MBIs face significant physical and emotional challenges that heighten their risk of postpartum depression and anxiety [26] and generally experience poorer postpartum mental health compared to that of singletons [27]. In turn, these have been associated with suboptimal breastfeeding outcomes [28]. The physical demands of frequent breastfeeding, combined with sleep deprivation and fatigue, exacerbate stress levels, which can negatively impact both maternal well-being and infant development [26,27].

Without adequate support, mothers face challenges that can lead to the early cessation of breastfeeding. Understanding MBI mothers’ experiences can enable health professionals to provide targeted parent support and education, potentially improving breastfeeding outcomes. Comprehensive guidelines that specifically address the unique challenges faced by mothers of MBIs are lacking, making it difficult for healthcare providers to offer consistent, specialised support. Qualitative research on the breastfeeding experiences of Australian mothers with MBIs is also scarce. This research aimed to provide insights into Australian mothers’ experiences of breastfeeding MBIs, including their perceived barriers and facilitators.

## 2. Materials and Methods

### 2.1. Study Design

Mothers of MBIs up to 24 months postpartum participated in an online anonymous survey using the secure web-based software platform designed to support data capture for research studies, Research Electronic Data Capture (REDCap, version 15.0.21), hosted at The University of Western Australia [29,30]. Qualitative and quantitative data were captured through survey items addressing breastfeeding barriers, facilitators, and experiences of MBI mothers. This mixed-methods design allowed for the collection of participants’ demographic, birth, and postpartum characteristics while also capturing the maternal voice, providing valuable insight into the lived experiences of mothers with MBIs. Reliability was not tested because the reliability of maternal recall regarding birth and breastfeeding events within 24 months postpartum is generally considered high [31,32].

### 2.2. Participants

Primiparous and multiparous participants living in Australia who were ≥18 years of age, could read English, and had given birth to MBIs within the last 24 months (to minimise recall bias) were recruited through social media posts on Australian Facebook pages targeting MBI parents (after receiving the page administrator’s approval). Ethics approval was granted by The University of Western Australia Human Research Ethics Committee (2024/ET000324; approved 2 May 2024). Participants provided digital consent, were reassured of anonymity, and were aware that they could exit the study at any time and for any reason. All data were securely stored in the Institutional Research Data Store (IRDS) to ensure confidentiality and privacy.

### 2.3. Data Collection

Participants completed an online 37-item questionnaire, which took approximately 10 min to complete. The questionnaire was developed with the engagement and input from consumers and experienced maternity care providers to ensure internal validity. Survey responses captured demographic data, including breastfeeding initiation, frequency, and duration; perceived challenges, barriers, and experiences; breastfeeding, expressions and infant formula supplementation characteristics (Supplementary Materials Survey Questions S1). Satisfaction with the accessed sources of professional support was rated using a Likert scale. Participants had the option of providing qualitative responses to the open-ended questions. The typed responses had no word limit.

### 2.4. Qualitative Data Analysis

Qualitative data were analysed using thematic analysis according to the Braun and Clarke framework [33]. Participants were asked three open-ended questions to identify the most helpful aspects of hospital care for breastfeeding multiples, areas for improvement, and their overall satisfaction with the care received during their hospital stay. Two researchers (MG and SP) independently read the responses to the questions multiple times to ensure familiarity with the data. They then identified key ideas for each question by labelling responses with codes, which were words or phrases that captured the meaning of the responses. The researchers then met to compare the findings and resolve any differences in coding; this process helped reduce bias and confirm that the codes were accurate. The generated codes were interpreted and developed into broader themes for each question. These themes were reviewed to ensure that they were clearly described, and concise names were developed for each theme. Finally, the findings are reported using relevant quotes to illustrate each theme.

### 2.5. Statistical Analysis

In this study, quantitative data are described as *n* (%) for categorical data and mean ± standard deviation (SD) or median [IQR] for continuous data. The Shapiro−Wilk Normality Test and Q-Q plot were used to assess normality of distribution.

Participants were recruited into two subgroups: *n* ≥ 30 primiparous and *n* ≥ 30 multiparous women, as preterm birth is more common in primiparous women [34], and breastfeeding and childcare experiences may also differ by parity [35]. For qualitative research, a sample size of *n* ≥ 30 is generally considered sufficient to achieve data saturation [36]. For the quantitative part of the survey, a sample size of 60 was determined using the ‘F tests–Linear multiple regression: Fixed model: R^2^ increase’ option in G*Power 3.1 [37]. Allowing two predictors (one for parity comparison), α = 0.05, and 56 participants (56 sample points = 28 participants × 2 parity groups) gave the study power of 0.80 to detect an effect size of 0.15. The required number of participants was increased to 60 to maintain the predicted power and address missing data issues.

Quantitative responses were analysed for outcome comparison by parity using Chi-square or Fisher’s exact tests, where appropriate. All analyses were performed using jamovi software [38]. Statistical significance was set at *p* < 0.05.

## 3. Results

### 3.1. Participants

**Figure 1:**
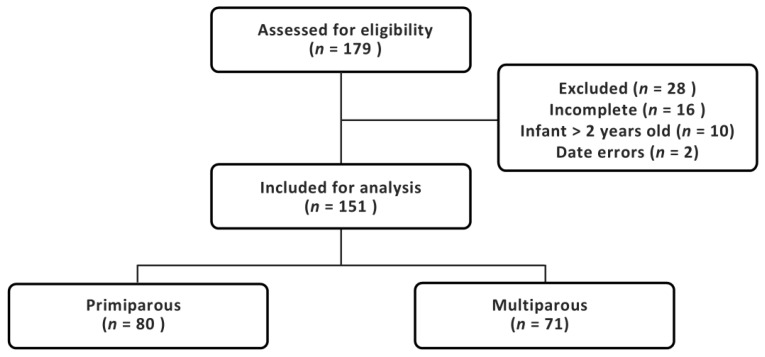
Study recruitment flowchart.

Participants were recruited from May to August 2024, with over-recruitment for both subgroups due to unforeseen high interest in the study. Of the 179 responses received, 151 met the study criteria (Figure 1). Most mothers (79.5%) identified as Australian (Table 1). A higher proportion of multiparous mothers had spontaneous vaginal births compared to primiparous mothers (14.1% vs. 3.8%, *p* = 0.024).

Mothers of twins accounted for most of the sample (98.7%), with 1.3% being mothers of triplets (Table 2). Primiparous mothers typically responded to the survey at a later postpartum time than multiparous mothers (*p* = 0.037). Over half of the multiparous women gave birth to their MBIs at term gestation, compared to one-third of the primiparous women, with most primiparous mothers giving birth at 32–36^+6^ weeks gestation.

### 3.2. Maternal and Infant Breastfeeding Characteristics

While similar proportions of primiparous and multiparous mothers planned to breastfeed their MBIs, significantly more primiparous mothers sought breastfeeding information during pregnancy (69.7%) compared to multiparous mothers (46.2%, *p* = 0.005) (Table 3).

Multiparous mothers were significantly more likely to initiate breastfeeding early, i.e., within an hour of birth (*p* = 0.004) (Table 4). The most common reasons for delayed initiation were newborn admission to the neonatal nursery (55.4%) and maternal health complications (38.5%). Almost all MBIs were breastfed/fed mother’s own milk (MOM) during their hospital stay, and nearly three-quarters of mothers were satisfied with the breastfeeding support provided during their hospital stay (Table 4).

Due to a software malfunction, a lower number of participants answered questions on breastfeeding patterns (Table 5), the use of lactation aids, reasons and frequency of breast expression (Table 6), and breastfeeding challenges (Table 7). Following discharge, most mothers both breastfed (77.3%) and expressed their milk (73%; Table 5). Commercial milk formula was typically introduced during the hospital stay. Overall, 53.4% of women reported fully breastfeeding their MBIs at some point during their breastfeeding experience, with an overall duration of those fully breastfeeding being ≤6 months. Primiparous mothers were significantly more likely than multiparous mothers to use scheduled feeds (*p* = 0.005) and not feed their infants at night (*p* = 0.009, Table 5), although they participated in the survey at later postpartum time (Table 2).

The use of lactation aids was common, with the use of electric breast pumps reported by almost all participants (Table 6). While the main reported reason for breast pump use was to boost milk supply (68.2%), it was also used to provide expressed breast milk feeds. Primiparous mothers had more prevalent nipple shield use (*p* = 0.005) and more frequent breast expressions (*p* = 0.016) to allow others to feed the infants breast milk (*p* = 0.007).

Overall, common breastfeeding concerns among mothers of MBIs did not differ by parity and included latching difficulties (56%), inadequate milk supply (49.3%), and sore nipples (46.7%; Table 7). Most mothers of preterm MBIs reported that preterm birth impacted breastfeeding due to the need for supplementary feeds (80.2%), latching difficulties, and the infants’ low energy levels (54.9% for both).

Regarding self-care and feeding management, significantly more primiparous mothers (79.5%) reported using a feeding and sleep schedule compared to multiparous mothers (60.7%, *p* = 0.019). The most highly rated self-care activities included staying hydrated (77.5%), social connection (60.5%), and light exercise (58.9%; Table 8).

**Figure 2:**
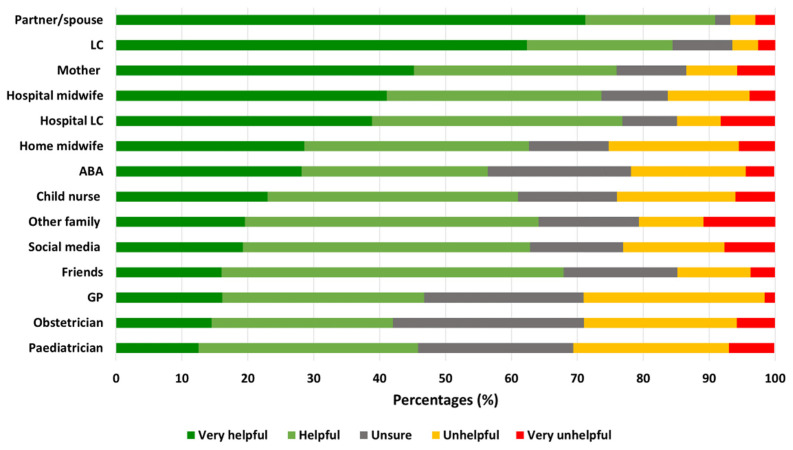
Helpfulness of clinical and community-based breastfeeding support. Data are presented as percentages. LC, lactation consultant; GP, general practitioner; ABA, Australian Breastfeeding Association.

Overall, most mothers rated their spouse/partner as the most helpful source of breastfeeding support, followed by lactation consultants and their own mothers. Paediatricians, obstetricians, and general practitioners were the least likely to be rated as helpful (Figure 2).

### 3.3. Qualitative Findings

**Figure 3:**
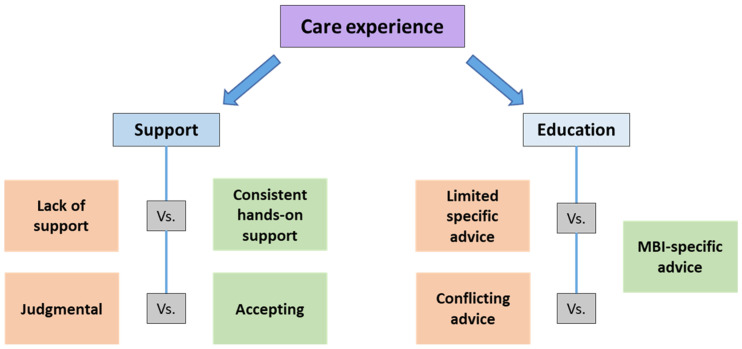
Women’s experiences of breastfeeding multiple birth infants.

One hundred and six mothers provided qualitative data, of whom 63 were primiparous. Reports of satisfaction with in-hospital breastfeeding support were provided by 69 mothers in this study. Primiparous mothers described more negative than positive care experiences. An inductive thematic analysis identified one main theme, ‘Care Experience’, which comprised two subthemes: ‘Support’ and ‘Education’, each containing opposing subcategories that highlight the differing nature of the care provided (Figure 3). Direct quotes are reported throughout the findings to further elucidate and provide evidence for the themes, with codes used to identify each participant’s parity, i.e., (*P*) = primiparous and (*M*) = multiparous.

#### 3.3.1. Subtheme 1: Support

The support subtheme reflected both positive and negative experiences of clinical breastfeeding support. The dichotomy of experiences underscored the variable quality of support provided and its impact on mothers during their postpartum hospital stay.

- ‘Lack of Support’ versus ‘Consistent Hands-on Support’

Primiparous mothers reported more negative care experiences, particularly feeling neglected due to the unavailability of staff or staff preoccupation with other tasks: *“Assistance and prompting from midwife attending after the birth, in my case they were more worried about completing paperwork and when they realised they forgot to feed the babies they gave my husband a bottle”. (P)*

Staffing issues and the busyness of the ward also impacted the care, with one mother reporting: *“There were too many agency nurses on shift when my babies were born, and I was handed a pump and told to express, but when I found it difficult, no one could spend any time to assist”. (P)*

Delays in assistance and lack of attention left mothers feeling unsupported during critical moments: *“I was not offered assistance in feeding them. When I asked for a lactation specialist or midwife to assist at times, it took hours, which meant the window had passed”. (P)*

Conversely, mothers valued when health professionals proactively assisted with breastfeeding initiation: *“I was very lucky and was able to breastfeed in the recovery room after my c-section with 2 midwives supporting each baby”. (P)*

Direct, hands-on assistance, such as helping with colostrum expression, breastfeeding, and pumping during the postpartum hospital stay, was perceived as highly beneficial:

*“The lactation consultants were incredible and visited me a few times to help teach me how to breastfeed my babies and how to make sure they latched properly. They also arranged a feeding plan for me to take home and trial when I was discharged”.*

*(P)*

*“A lactation consultant checked in with me almost daily plus midwives assisted in manually helping my milk to come in after an induced birth and c section”.*

*(M)*

*“I had a wonderful midwife who helped my husband and I with how to collect colostrum”.*

*(P)*

- ‘Judgemental’ versus ‘Accepting’

Mothers reported feeling coerced or judged by healthcare professionals regarding their breastfeeding practices: *“I was pressured to only breastfeed them both at the same time rather than focus on each baby. It was very difficult to breastfeed premmie babies together”. (P)*

Some staff were inflexible in their advice, making mothers feel criticised rather than supported: *“Less judgement from the midwives would have been helpful. some are so forceful with their opinions”. (P)* Another shared a similar view: *“I found the midwives and some lactation consultants outdated and forceful when I struggled to breastfeed”. (P)*

In one case, feelings of shame negatively impacted a mother’s confidence and overall breastfeeding experience: *“Lactation support in a private hospital directly impacted my ability to feed. I was judged, unsupported and publicly humiliated by the team”. (P)*

Conversely, mothers valued healthcare professionals who were understanding, non-judgemental, and supportive of their feeding decisions: *“I appreciated it when nurses or consultants were flexible and did not guilt me for using formula. I found health care providers who really listened to my concerns and were more hands on with their assessment most helpful”. (P).*

In reporting how care could be improved, another mother emphasised the need for *“Professionals being flexible and understanding how hard breastfeeding is, the additional challenges with feeding twins and not demonising formula”. (M)* Additionally, there were calls for *“more empathy” (M)* and *“less judgement and more support”. (M)*

#### 3.3.2. Subtheme 2: Education

Breastfeeding education specific to MBIs was effective in preparing mothers to breastfeed their MBIs during their hospital stay and after discharge at home; however, not all mothers reported access to MBI-specific education.

- ‘MBI-specific Advice’ versus ‘Limited Specific Advice’ and ‘Conflicting Advice’

MBI-specific support from health professionals was essential for establishing and maintaining successful breastfeeding practices: *“Visiting lactation nurse was very helpful at showing how to tandem feed”. (P)*, while an international board-certified lactation consultant (IBCLC) provided essential guidance on *“how important it was to pump regularly to establish supply. She also assessed my twins’ latch and positions and helped optimise things so that they would feed better”. (P)*

Not surprisingly, a common concern was the lack of specialised information and guidance on breastfeeding multiples with primiparous mothers reporting it more often than multiparous mothers: *“Very limited knowledge on how to support twin feeding despite being the main hospital in the region for multiples”. (P)* and *“There is a lack of education on feeding multiples in midwifery training. As a midwife myself I am very disappointed in this gap”. (M)* Many mothers felt that the support they received was primarily for singletons: *“Needed more advice and practice on tandem breastfeeding. Most support was centred around breastfeeding a single baby”. (P)*

Mothers often received conflicting advice from different healthcare professionals, which created confusion and stress: *“Incorrect advice given regarding expressing equipment sizing causing damage”. (P)* Another added: *“All the different LCs said different things. There were good ones which worked with me, and bad ones who told me to do really strange things that weren’t actually a good idea”. (M)* The lack of consistent advice hindered their ability to make informed breastfeeding decisions: *“… there was a huge variety of advice and opinions, so it was hard to consolidate everything”. (P)*

#### 3.3.3. Maternally Identified Areas of Improvement

**Figure 4:**
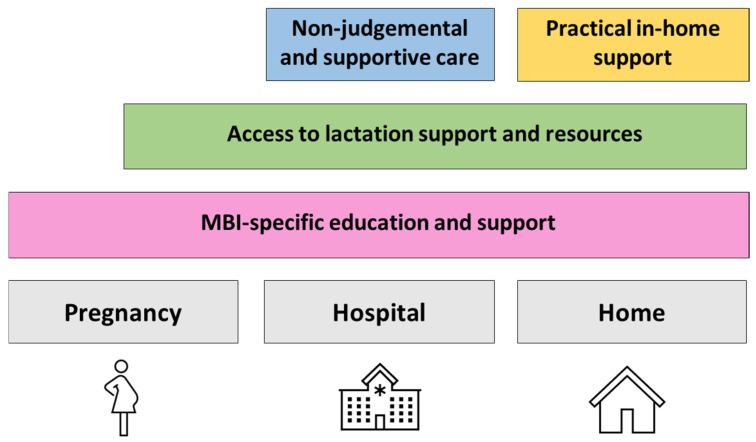
Identified areas for improvement in breastfeeding support for families with multiple birth infants (MBI).

Figure 4 illustrates the maternally identified areas of improvement to support MBI feeding, which included MBI-specific education and support, practical in-home support, access to lactation support and resources, and non-judgemental and supportive care.

- MBI-specific education and support

Mothers expressed a need for education and support that is specific to the unique challenges of breastfeeding MBIs, as outlined in Section 3.3.2.

- Non-judgemental and supportive care

Non-judgemental and supportive care from health providers helped mothers feel valued and supported in their feeding journeys, as illustrated by the quotes in Section 3.3.2.

- Practical in-home support

Mothers identified the need for in-home support, such as help with chores and having people available to help with feeds: *“Multiple mums need an extra pair of hands to do literally anything. Help with feeds is crucial. If my own mother didn’t live with us for seven months, there’s no way I could have kept up with feeding them as long as I did”. (P)*

Regular check-ups at home and guidance from health professionals were suggested including *“Additional home visits from maternal child health nurses or appointments with them” (P)* and *“Free support from midwives/lactation consultants in the home until babies are 4 months of age”. (M)*

- Access to lactation support

Reliable access to lactation consultants and lactation aids, as well as support for expressing milk, were reported as crucial for successful breastfeeding. Suggestions included *“free or subsidised certified lactation consultant visits especially in the early days to help set up a way to feed that works for them at home. Subsidised breastfeeding pillows etc.” (M)*, and *“ensure that women with babies in the NICU (neonatal intensive care unit) are given a pump asap and taught to use it if their health is permitting”. (P)*

## 4. Discussion

**Figure 5:**
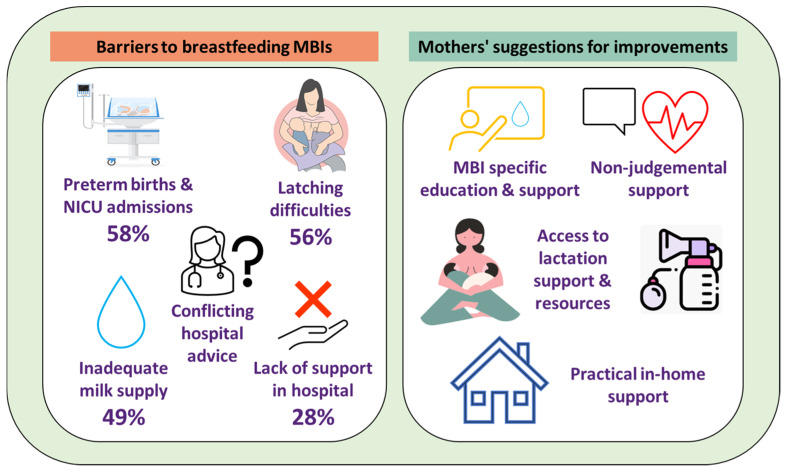
Barriers to breastfeeding multiple birth infants (MBIs) and areas for improvement in breastfeeding support for multiple birth families, as reported by MBI mothers.

This study highlighted the lived breastfeeding experiences and diverse needs of mothers of MBIs. The findings showed significant differences in the experiences of primiparous and multiparous mothers, with the former expressing more dissatisfaction with breastfeeding MBIs support and guidance. Additionally, the study emphasised the importance of tailored antenatal education, practical support systems, and policy initiatives to improve breastfeeding outcomes for MBI families (Figure 5).

Common breastfeeding challenges reported by all mothers included latching difficulties (56%), concerns about milk supply (49%), and nipple pain (25%; Table 7 and Figure 5). Low milk supply is one of major impediments to breastfeeding, leading to early formula supplementation and cessation of breastfeeding [19,20], potentially affecting mothers of MBIs on a greater scale. There are a limited number of human milk banks in Australia that provide pasteurised donor human milk to preterm infants born at <32 weeks gestation (up until they reach 34 weeks corrected gestational age). At this stage, not all NICUs have access to pasteurised donor human milk.

A large proportion of mothers relied on breast pumps (99%) and nursing pillows (89%; Table 6). This reflects the importance of and the need for better access to lactation aids, such as hospital-grade pumps, to sustain breastfeeding and improve breastfeeding outcomes in this population, especially when direct feeding is challenging due to preterm births, latching issues, and positioning. The majority of MBIs are born preterm, and more than half of the infants in this study were admitted to the NICU or intermediate care nursery for newborn care. It can be extrapolated that some infants, therefore, lacked the sucking skills and stamina needed to adequately and frequently remove colostrum and milk from the breast, which can persist in the weeks after birth [39,40]. The frequent use of an electric breast pump after the birth of a sick or preterm infant is important for establishing adequate milk production [41,42,43,44]. Research has demonstrated the efficacy of electric breast pumps in removing milk from the breast, and they are recommended for partially and fully pump-dependent mothers [45,46]. Antenatal education could include information on where to source effective hospital-grade pumps and other necessary lactation aids, along with training on their use, to prepare families for the unique challenges of establishing lactation and breastfeeding for two or more infants, who are often born preterm.

While there is a range of maternity care options in Australia that vary in terms of continuity of care, all options would include access to a midwife during pregnancy and the immediate postpartum period. During routine pregnancy care, the midwifes typically lead antenatal discussions on breastfeeding and advise resources such as breastfeeding classes. After birth, midwives provide direct assistance and information during the postpartum hospital stay, and IBCLCs are available for inpatient support in many maternity wards. Beyond discharge, many hospitals provide home-visiting midwifery services, so some in-home lactation care is available. There is limited availability of publicly funded lactation clinics in the community staffed by IBCLCs or midwives with advanced skills and knowledge in breastfeeding support. Further, there is a peer-support organisation, the Australian Breastfeeding Association (ABA), that provides a federally funded 24 h telephone helpline, a website [47], and local support groups where face-to-face support is provided by trained voluntary breastfeeding counsellors. Private IBCLCs can also be accessed in a clinic or through in-home consultations with a fee-for-service model.

Although most mothers (72%, Table 4) expressed satisfaction with the in-hospital breastfeeding support, the qualitative data indicated that primiparous mothers were more likely to express dissatisfaction than multiparous mothers, often citing inconsistent advice, lack of hands-on support, and unpreparedness for the realities of feeding multiples. This dissatisfaction may be linked to their lack of prior experience, higher rates of preterm birth (Table 2), and associated challenges, all of which can disrupt early breastfeeding opportunities and increase their education and support needs. While both primiparous and multiparous mothers had similar intentions to breastfeed, their experiences diverged in several ways. Primiparous mothers were more likely to seek breastfeeding information during pregnancy yet less likely to initiate breastfeeding within the first hour after birth than multiparous mothers (Table 4). Early and frequent milk removal is critical for developing an adequate milk supply, particularly in the first two weeks postpartum, as regular stimulation signals the body to continue producing milk [48]. Research indicates that establishing a robust supply by two weeks postpartum is predictive of longer-term milk production [49]. Given that MBI mothers must produce milk for multiple infants, inadequate guidance during this crucial period can significantly impact breastfeeding outcomes. Preterm birth, which is common among MBIs, introduces additional challenges due to infants’ underdeveloped sucking reflexes and oro-facial muscles, which can impair effective milk transfer [50,51]. This can lead to insufficient milk removal and reduced supply if alternative methods, such as breast expression, are not consistently used. Primiparous mothers, in particular, reported that the delayed initiation of breastfeeding was due to their infants’ admission to the neonatal nursery (Table 4). This delay limits immediate skin-to-skin contact and direct breastfeeding opportunities, both of which are crucial for establishing milk production and supporting early bonding [52].

The qualitative data further highlighted differences between primiparous and multiparous mothers in their experiences and perceptions of support. While many mothers expressed feelings of unpreparedness for challenges such as preterm birth complications, tandem feeding, and the intensive time commitment required for breastfeeding MBIs, primiparous mothers frequently reported receiving conflicting advice and judgemental attitudes towards feeding decisions, which contributed to confusion and self-doubt regarding their ability to breastfeed (Section 3.3.1, Subtheme 1: Support). In contrast, multiparous mothers reported more positive experiences with hospital staff, were more likely to initiate breastfeeding earlier (Table 4), and were less likely to have scheduled feeds, express breast milk frequently, and use a nipple shield (Table 6). Prior positive breastfeeding experiences contribute significantly to maternal knowledge and confidence [53], which aids in navigating the challenges of breastfeeding multiples. Given that first-time mothers have greater learning needs, targeted interventions could provide detailed, practical information necessary to prepare them for the realities of breastfeeding multiples. This may include additional in-hospital lactation consultations, follow-up home visits, and peer-support programmes to reinforce consistent supportive guidance. Rather than simply setting expectations, antenatal education should focus on equipping mothers with clear, evidence-based information about the complexities of breastfeeding multiples. Training should also emphasise respect and support for mothers’ feeding choices while ensuring they receive individualised care [54].

Overall, mothers expressed the need for MBI-specific breastfeeding advice that acknowledges the increased workload and challenges of feeding multiples (Figure 4). While existing breastfeeding guidelines are based on evidence supporting the health benefits of human milk for both infants and mothers, regardless of singleton or multiple births, they do not account for the practical difficulties unique to mothers of MBIs. Mothers found that breastfeeding education classes primarily focused on term and singleton birth infants, leaving them unprepared for the complexities of feeding multiples. This highlights a gap in breastfeeding support, rather than in the evidence itself. Developing comprehensive, MBI-specific antenatal breastfeeding education programmes could help bridge this gap by addressing the logistical challenges of managing feeding schedules, tandem feeding techniques, and efficient pumping routines. Such education could improve maternal self-efficacy and better prepare mothers for the realities of breastfeeding multiple infants [55].

Support systems play a pivotal role in breastfeeding success among mothers with MBIs. Research indicates that strong partner and family support is associated with longer breastfeeding duration [56]. The mothers in this study cited their partners and their own mothers as the most helpful sources of support (Figure 2), particularly in terms of providing hands-on assistance with feeding and household tasks. Many expressed that practical in-home support, such as help with chores, childcare, or feeding assistance, would significantly alleviate their burden. Previous qualitative research on Australian MBI mothers also highlights the significant emotional and physical demands placed on mothers of multiples when breastfeeding, emphasising the need for external support systems, such as LCs and family involvement, to help manage the intensive nature of feeding multiple infants [57]. A model for such support exists in New Zealand, where the government provides 240 h of non-means-tested family support services within 12 months of birth for families of twins and 1560 h within 24 months for families of triplets or more. This support includes cleaning, cooking, and laundry services [58]. The implementation of similar initiatives in Australia could be transformative, allowing mothers to focus on breastfeeding rather than household management. Expanding access to practical in-home support, including assistance with tandem feeding and expressing, may significantly improve breastfeeding confidence and alleviate stress in first-time mothers of MBIs.

The Australian Multiple Birth Association (AMBA), a key not-for-profit organisation, provides free social services to help families with MBIs [59]. Mothers cited AMBA as an important source of MBI-specific information, where they could connect with other mothers who offered peer support based on their own MBI experiences. The availability of community-based peer support has been shown to enhance breastfeeding outcomes, with mothers more likely to initiate breastfeeding, exclusively breastfeed, and breastfeed for longer durations [60]. Additionally, access to professional lactation support remains critical. While our study found that most mothers did not cite cost as a barrier to accessing private lactation support (Table 7), this likely reflects the relatively high socioeconomic status of our sample population. Face-to-face support has undeniable benefits when it comes to breastfeeding support [61]; however, for many women, the cost of private services remains a significant obstacle.

Regarding maternal self-care, the most rated self-care activities were staying hydrated (78%), having social connections (61%), and engaging in light exercise (60%; Table 8). While increasing fluid intake during lactation is unlikely to increase milk production, it is crucial for breastfeeding women to compensate water loss and prevent dehydration [62,63,64], and our participants recognised staying hydrated as a key self-care strategy. Importantly, more than half of the mothers of MBIs reported engaging in light exercise, which is a considerable step forward, as only 23% of Australian mothers of singletons meet the postpartum exercise recommendations of 150 min/week [65].

This study is one of the few that examined both breastfeeding characteristics and experiences of Australian MBI mothers. Another strength of this study is the relatively high survey response rate, which reflects the importance of this topic to mothers of MBIs. Despite the software malfunction and lower response rate, the minimum sample size required to assess differences between primiparous and multiparous women was achieved in the majority of responses to questions on infant feeding characteristics, lactation aids, and breastfeeding concerns (Table 5, Table 6 and Table 7).

This study has several limitations. Regardless of the importance of the survey focus, the longer the survey, the higher the risk of respondent fatigue, which may result in poor or inconsistent data; therefore, we were not able to address all aspects of breastfeeding MBIs. The overwhelming majority of the study participants were mothers of twins, limiting the applicability of the findings to mothers of triplets or higher-order multiples, who may face additional or different challenges. Addressing this limitation is difficult, as the prevalence of higher-order multiple births is low, and the additional demands of caring for three or more infants likely make it harder for parents to participate in research. The study participants predominantly identified as Australian and were from higher socioeconomic backgrounds, which may have biased the results. Financial resources can greatly influence access to lactation aids and/or support, meaning that the findings may not fully represent the experiences of culturally and linguistically diverse and disadvantaged populations within Australia. However, we did capture Aboriginal and/or Torres Strait Islander people (3.3%), which represent 3.2% of the total Australian population [66].

Overall, the study’s findings highlight the significant challenges mothers of MBIs face in breastfeeding, underscoring the need for MBI-specific support, tailored guidelines, and improved access to consistent and practical care. Future studies could focus on mothers of higher-order multiples and explore the socioeconomic and cultural influences on breastfeeding experiences in MBI mothers. Given the homogeneity of the current sample, studies with more diverse populations could uncover different challenges and support needs, informing more inclusive and culturally sensitive breastfeeding support strategies. Future research could also include an examination of midwives’, neonatal nurses, and community child health nurses’ challenges and learning needs in relation to their care of MBI families. Furthermore, a quantitative approach, such as measuring 24 h milk production and infants’ intake of breast milk and/or commercial milk formula, as well as feeding and expressing dynamics, would be prudent. These perspectives can inform health professional education and uncover strategies for enhancing MBI breastfeeding support.

## 5. Conclusions

While full breastfeeding for six months may be challenging for many mothers of MBIs due to inherent obstacles, targeted support and tailored interventions can play a crucial role in improving breastfeeding outcomes. Addressing the unique barriers faced by mothers of MBIs can help create a more empowering environment that allows mothers to sustain breastfeeding, leading to better health and well-being for both mothers and their infants.

## Figures and Tables

**Table 1:** Maternal characteristics.

	Overall *n* = 151	Primiparous *n* = 80	Multiparous *n* = 71	*p*-Value ^3^
Cultural/ethnic group ^2^				
Australian	120 (79.5) ^1^	64 (80)	56 (78.9)	0.86
British	16 (10.6)	5 (6.25)	11 (15.5)	0.066
Asian	11 (7.3)	7 (8.75)	4 (5.6)	0.46
Aboriginal	5 (3.3)	1 (1.25)	4 (5.6)	0.14
Other	15 (9.9)	10 (12.5)	5 (7.0)	0.24
SEIFA index percentile	65 [43, 81]	60.5 [41, 79.75]	70 [49, 84]	0.14
0–30, most disadvantaged	20 (13.2)	11 (13.75)	9 (12.7)	
30–70,	67 (44.4)	40 (50.0)	27 (38.0)	
70–100, least disadvantaged	64 (42.4)	29 (36.25)	35 (49.3)	
Birth mode				
Elective CS	78 (51.7)	45 (56.25)	33 (46.5)	0.23
Non-elective CS	43 (28.5)	22 (27.5)	21 (29.6)	0.78
Spontaneous vaginal	13 (8.6)	3 (3.75)	10 (14.1)	**0.024**
Assisted vaginal	10 (6.6)	8 (10)	2 (2.8)	0.076
Breech vaginal	7 (4.6)	2 (2.5)	5 (7.0)	0.19
Parity		1	2 [2, 3]	
Previous breastfeeding duration (months)				
Child 1			13 [12, 18]	
Child 2			12 [10.5, 15.5]	

^1^ Data are presented as mean ± SD, median [IQR], or *n* (%); ^2^ sum of percentages > 100% as women could select more than one ethnic group. ^3^ *p*-value indicates a significant difference between the primiparous and multiparous groups using the Chi-square or Fisher’s exact test, where appropriate. Bold font indicates a significant difference. SEIFA, Socio-Economic Indexes for Australia (categorises postal area codes by relative socioeconomic advantage and disadvantage). CS, caesarean section.

**Table 2:** Infant characteristics.

	Overall *n* = 151	Primiparous *n* = 80	Multiparous *n* = 71	*p*-Value ^2^
Multiple birth categories				0.93
Twins	149 (98.7) ^1^	79 (98.75)	70 (98.6)	
Triplets	2 (1.3)	1 (1.25)	1 (1.4)	
Birth gestation (weeks)	36.0 [34.0, 37.0]	36.1 [34.4, 37.1]	37.0 [34.8, 37.5]	**0.019**
Gestational age category				
Extremely preterm (<28 weeks)	1 (0.7)	0 (0.0)	1 (1.4)	0.47
Very preterm (28–31^+6^ weeks)	12 (7.9)	8 (10)	4 (5.6)	0.32
Moderate to late preterm (32–36^+6^)	75 (49.7)	46 (57.5)	29 (41)	**0.041**
Term (≥37)	63 (41.7)	26 (32.5)	37 (52.1)	**0.015**
MBI age at time of survey (months)	9.1 [4.1, 15.8]	10.4 [5.8, 17.3]	7.5 [3.5, 5.9]	**0.037**

^1^ Data are presented as median and interquartile range [IQR] or *n* (%). ^2^ *p*-value indicates a significant difference between the primiparous and multiparous groups using the Chi-square or Fisher’s exact test, where appropriate. Bold font indicates a significant difference. MBI, multiple birth infants.

**Table 3:** Antenatal breastfeeding intentions and access to breastfeeding information.

	Overall *n* = 151	Primiparous *n* = 80	Multiparous *n* = 71	*p*-Value ^2^
Intended to breastfeed				0.72
Yes	123 (87.2) ^1^	66 (86.8)	57 (87.7)	
No	5 (3.5)	2 (2.6)	3 (4.6)	
Unsure	13 (9.2)	8 (10.5)	5 (7.7)	
Accessed breastfeeding information	83 (58.9)	53 (69.7)	30 (46.2)	**0.005**

^1^ Data are presented as *n* (%). ^2^ *p*-value indicates a significant difference between the primiparous and multiparous groups using the Chi-square or Fisher’s exact test, where appropriate. Bold font indicates a significant difference.

**Table 4:** Breastfeeding initiation and satisfaction with hospital-based breastfeeding support.

	Overall	Primiparous	Multiparous	*p*-Value ^3^
Early breastfeeding initiation ^2^	***n* = 129**	***n* = 69**	***n* = 60**	
Yes	64 (49.6) ^1^	26 (37.7)	38 (63.3)	**0.004**
Reasons for late initiation of breastfeeding	*n* = 65	*n* = 43	*n* = 22	
Neonatal unit admission	36 (55.4)	21 (48.8)	15 (68.2)	0.14
Mother unwell	25 (38.5)	18 (27.7)	7 (31.8)	0.43
Could not express milk	2 (3.1)	2 (4.7)	0 (0.0)	0.55
Infant/s not interested	1 (1.5)	1 (2.3)	0 (0.0)	1.00
Other	1 (1.5)	1 (2.3)	0 (0.0)	1.00
Infant/s fed MOM	*n* = 141	*n* = 76	*n* = 65	
Yes	134 (95.0)	72 (94.7)	62 (95.4)	0.86
Satisfied with breastfeeding support	*n* = 141	*n* = 76	*n* = 65	
Yes	101 (71.6)	51 (67.1)	50 (76.9)	0.20

^1^ Data are presented as *n* (%). ^2^ Includes breastfeeding and/or providing breast milk to the infants. ^3^ *p*-value indicates a significant difference between the primiparous and multiparous groups using the Chi-square or Fisher’s exact test, where appropriate. Bold font indicates a significant difference. MOM, mother’s own milk.

**Table 5:** Infant feeding characteristics.

	Overall	Primiparous	Multiparous	*p*-Value ^4^
Home feeding mode ^2^	***n* = 141**	***n* = 76**	***n* = 65**	
MOM via breastfeeding	109 (77.3) ^1^	54 (71)	55 (84.6)	0.055
Expressed breast milk	103 (73.0)	57 (75.0)	46 (70.8)	0.57
Commercial milk formula	80 (56.7)	40 (52.6)	40 (61.5)	0.29
Donor human milk	4 (2.8)	2 (2.6)	2 (3.1)	0.87
Currently breastfeeding	*n* = 129	*n* = 73	*n* = 56	
Yes	68 (52.7)	34 (46.6)	34 (60.7)	0.11
Time of breastfeeding cessation (months)	*n* = 53	*n* = 34	*n* =19	
Median [Q1, Q3]	5.0 [3, 9]	5.0 [3.75, 9]	4.0 [2, 11]	0.62
Fully breastfeeding status	*n* = 116	*n* = 65	*n* = 51	
Ever fully breastfed ^3^	62 (53.4)	37 (56.9)	25 (49.0)	0.40
Never fully breastfed	40 (34.5)	21 (32.3)	19 (37.3)	0.58
Breastfeeding frequency/24 h				
Twin 1	5.0 [3, 7]	5.0 [2, 7]	6.0 [4, 7]	0.18
Twin 2	5.0 [3, 7]	4.0 [2, 7]	6.0 [3.75, 7.25]	0.084
Breastfeeding pattern	*n* = 59	*n* = 29	*n* = 30	
Same breastfeeding pattern	33 (56.0)	16 (55.2)	17 (56.7)	0.91
One infant feeds more efficiently	21 (35.6)	12 (41.4)	9 (30.0)	0.36
One infant sucks more strongly	16 (27.1)	11 (38.0)	5 (16.7)	0.066
One infant feeds more frequently	10 (17.0)	6 (20.7)	4 (13.3)	0.45
Management of night feeds	*n* = 129	*n* = 73	*n* = 56	
Feed infants together	56 (43.4)	33 (45.2)	23 (41.1)	0.64
Feed each infant to demand	49 (38.0)	23 (31.5)	26 (46.4)	0.083
No night feeds	36 (27.9)	27 (37.0)	9 (16.1)	**0.009**
Scheduled feeds	17 (13.2)	15 (20.6)	2 (3.6)	**0.005**
Formula introduced (days)	0.91 [0.91, 60.9]	15.2 [0.91, 68.5]	0.91 [0.91, 30.4]	0.097
Food introduced (months)	*n* = 129	*n* = 73	*n* = 56	
Solid foods	6.0 [5, 6]	6.0 [5, 6]	6.0 [6, 6]	0.073
Cow’s milk	12.0 [11, 12]	12.0 [11.5, 12]	12.0 [5.75, 12]	0.11

^1^ Data are presented as mean ± SD, median [IQR], or *n* (%). ^2^ Sum of percentages > 100% as participants could select more than one response. ^3^ Fully breastfed at some point during their breastfeeding experience, as reported by mothers. ^4^ *p*-value indicates a significant difference between the primiparous and multiparous groups using the Chi-square or Fisher’s exact test, where appropriate. Bold font indicates a significant difference. MOM, mothers’ own milk.

**Table 6:** Lactation aid use, reasons, and frequency of breast expression.

	Overall	Primiparous	Multiparous	*p*-Value ^3^
Lactation aids ^2^	***n* = 74**	***n* = 45**	***n* = 29**	
Electric breast pump	73 (98.7) ^1^	45 (100)	28 (96.6)	0.21
Nursing pillow	66 (89.2)	39 (86.7)	27 (93.1)	0.38
Nipple shields	33 (44.6)	26 (57.8)	7 (24.1)	**0.005**
Silicone milk catcher	20 (27.0)	14 (31.1)	6 (20.7)	0.32
Domperidone	15 (20.3)	10 (22.2)	5 (17.2)	0.29
Other galactagogues	29 (39.2)	19 (42.2)	10 (34.5)	0.60
Manual breast pump	9 (12.2)	7 (15.6)	2 (6.9)	0.27
Nursing supplementer	1 (1.4)	0 (0)	1 (3.5)	0.39
Reasons for breast milk expression	*n* = 135	*n* = 73	*n* = 62	
Increase milk supply	92 (68.2)	47 (64.4)	45 (72.6)	0.31
Allow others to feed	88 (65.2)	55 (75.3)	33 (53.2)	**0.007**
Obtain EBM for top-ups	85 (63.0)	44 (60.3)	41 (66.1)	0.48
Infant did not latch	59 (43.7)	35 (48.0)	24 (38.7)	0.28
Poor latch and/or nipple pain	39 (28.9)	20 (27.4)	19 (30.7)	0.68
Relieve engorgement	29 (21.5)	16 (21.9)	13 (21.0)	0.89
Relieve pain from breast conditions	18 (13.3)	8 (11.0)	10 (16.1)	0.38
Expression frequency	*n* = 63	*n* = 37	*n* = 26	
Few times per week	9 (14.3)	2 (5.4)	7 (26.9)	**0.016**
Once a day	4 (6.3)	2 (5.4)	2 (7.7)	0.71
2–3 times per day	20 (31.7)	14 (37.8)	6 (23.1)	0.22
4–5 times per day	9 (14.3)	8 (21.6)	1 (3.8)	**0.047**
≥6 times per day	21 (33.3)	11 (29.7)	10 (38.5)	0.47

^1^ Data are presented as *n* (%); ^2^ sum of percentages > 100% as participants could select more than one response. EBM, expressed breast milk. ^3^ *p*-value indicates a significant difference between the primiparous and multiparous groups using the Chi-square or Fisher’s exact test, where appropriate. Bold font indicates a significant difference.

**Table 7:** Breastfeeding challenges.

	Overall *n* = 91	Primiparous *n* = 59	Multiparous *n* = 32	*p*-Value ^4^
Preterm birth impacts on breastfeeding				
Yes	70 (76.9) ^1^	47 (79.7)	23 (71.9)	0.40
Preterm aspects affecting breastfeeding ^2^				
Supplementary feeds	73 (80.2)	45 (76.3)	28 (87.5)	0.20
Latching difficulty	50 (54.9)	34 (57.6)	16 (50)	0.49
Lack of energy	50 (54.9)	30 (50.8)	20 (62.5)	0.29
Poor sucking reflex	46 (50.5)	30 (50.8)	16 (50)	0.94
Prolonged separation	40 (43.9)	25 (42.4)	15 (46.9)	0.68
Poor suck/swallow coordination	32 (35.2)	25 (42.4)	7 (21.9)	0.051
No impact on breastfeeding	10 (11)	8 (13.6)	2 (6.3)	0.29
Breastfeeding concerns ^3^	*n* = 75	*n* = 46	*n* = 29	
Latching difficulty	42 (56.0)	25 (54.4)	17 (58.6)	0.72
Low milk supply	37 (49.3)	22 (47.8)	15 (51.7)	0.74
Sore nipples	35 (46.7)	20 (43.5)	15 (51.7)	0.49
Positioning difficulties	31 (41.3)	21 (45.7)	10 (34.5)	0.34
Long feeding duration	31 (41.3)	23 (50.0)	8 (27.6)	0.055
Damaged nipples	19 (25.3)	9 (19.6)	10 (34.5)	0.15
Blocked ducts	19 (25.3)	13 (28.3)	6 (20.7)	0.46
Mastitis	13 (17.3)	11 (24.0)	2 (7.0)	0.068
Oversupply	10 (13.3)	9 (19.6)	1 (3.5)	0.050
No concerns	5 (6.7)	3 (6.5)	2 (7.0)	0.96
Other	8 (10.7)	3 (6.5)	5 (17.24)	0.14
Did cost of lactation support influence access?	*n* = 109	*n* = 61	*n* = 48	
Significantly	17 (15.6)	11 (18.0)	6 (12.5)	0.33
Somewhat	23 (21.1)	14 (23.0)	9 (18.75)	0.44
No	69 (63.3)	36 (59.0)	33 (68.75)	0.53

^1^ Data are presented as *n* (%); ^2^ the sum of percentages > 100% as participants could select more than one response. ^3^ General breastfeeding concerns, including mothers of term infants. ^4^ *p*-value indicates a significant difference between the primiparous and multiparous groups using the Chi-square or Fisher’s exact test, where appropriate.

**Table 8:** Maternal self-care activities during the first year postpartum.

	Overall *n* = 129	Primiparous *n* = 73	Multiparous *n* = 56	*p*-Value ^3^
Used feeding and sleep schedule				
Yes	92 (71.3) ^1^	58 (79.5)	34 (60.7)	**0.019**
Self-care activities in first 12 months ^2^				
Staying hydrated	100 (77.5)	53 (72.6)	47 (83.9)	0.13
Social connection	78 (60.5)	43 (58.9)	30 (53.6)	0.55
Light exercise	76 (58.9)	43 (58.9)	33 (58.9)	1.0
Balanced diet	69 (53.5)	42 (57.5)	27 (48.2)	0.29
Taking breaks	46 (35.7)	26 (35.6)	20 (35.7)	1.0
Enjoyable activities	40 (31.0)	24 (32.9)	16 (28.6)	0.60
Stress management	18 (14.0)	13 (17.8)	5 (8.9)	0.15
None	7 (5.4)	6 (8.2)	1 (1.8)	0.11
Other	2 (1.6)	1 (1.4)	1 (1.8)	1.00

^1^ Data are reported as *n* (%); ^2^ sum of percentages > 100% as participants could select more than one response. ^3^ *p*-value indicates a significant difference between the primiparous and multiparous groups using the Chi-square or Fisher’s exact test, where appropriate. Bold font indicates a significant difference.

## Data Availability

Restrictions apply to the availability of some or all data generated or analysed during this study. The corresponding author will, on request, detail the restrictions and any conditions under which access to some data may be provided.

## Supplementary Materials

The following supporting information can be downloaded at: https://www.mdpi.com/article/10.3390/nu17101669/s1, Survey Questions S1: Online Survey.

## Abbreviations

The following abbreviations are used in this manuscript:

α	Alpha
ABA	Australian Breastfeeding Association
AMBA	The Australian Multiple Birth Association
CS	Caesarean section
EBM	Expressed breast milk
GP	General practitioner
IBCLC	International board-certified lactation consultant
IRDS	Institutional Research Data Store
LC	Lactation consultant
MBI	Multiple birth infants
MOM	Mother’s own milk
NICU	Neonatal intensive care unit
PIMS	Perceived insufficient milk supply
REDCap	Research Electronic Data Capture
SEIFA	Socio-Economic Indexes for Australia
UWA	The University of Western Australia
WHO	World Health Organization
