# Supplementary material for: Breastfeeding Experiences in Australian Mothers of Multiple Birth Infants
Source: Nutrients. 2025 May 14;17(10):1669. doi: 10.3390/nu17101669 (PMC12113776; doi:10.3390/nu17101669)
Supplement: Supplementary file 1 [file nutrients-17-01669-s001.zip › nutrients-3608649-supplementary.pdf]

nutrients

Breastfeeding Experiences in Australian Mothers of Multiple Birth Infants

Muja A. Gama [1,2,3,4], Jacki L. McEachran [1,2,3], Ashleigh H. Warden [1,2,3], Demelza J. Ireland [4], Donna T. Geddes [1,2,3], Sharon L. Perrella [1,2,3] and Zoya Gridneva [1,2,3,*]

1 School of Molecular Sciences, The University of Western Australia, Crawley, WA 6009, Australia; gamamuja@gmail.com (M.A.G.); ashleigh.warden@uwa.edu.au (A.H.W.); jacki.mceachran@uwa.edu.au (J.L.M.); donna.geddes@uwa.edu.au (D.T.G.) sharon.perrella@uwa.edu.au (S.L.P.)
2 ABREAST Network, Perth, WA 6000, Australia
3 UWA Centre for Human Lactation Research and Translation, Crawley, WA 6009, Australia
4 School of Biomedical Sciences, The University of Western Australia, Crawley, WA 6009, Australia; demelza.ireland@uwa.edu.au (D.J.I.)
* Correspondence: zoya.gridneva@uwa.edu.au (Z.G.)

Online Survey questions

1. **Are your multiple birth babies over the age of 2 years?**
   - Yes
   - No

   If answered Yes Drop-down window:
   What is the date of birth of your first-born multiple birth baby?

<u>DEMOGRAPHICS</u>

2. **To which ethnic group/s do you belong to? (Tick all that apply)**
   - Aboriginal or Torres Strait Islander
   - Australian
   - Pacific Islander
   - Asian
   - British
   - North-West European
   - South-East European
   - Sub-Saharan African
   - Central or South American
   - Other – please specify (provide text box to answer)

3. **Please provide your current postal code**

4. **Are you a first-time mother?**
   - Yes
   - No

   If answered No ask Q5

5. **If no, besides your multiple birth babies, how many other children do you have?** (Drop box list of 1-5 and more than 5)

6. **Did you breastfeed your other children? If yes, how long did you breastfeed Child 1? Child 2? Child 3? (months) (Type answer)**

<u>BIRTH AND INFANT DETAILS</u>

7. **How many multiple birth babies did you give birth to? E.g., 2 = twins, 3 = triplets**
   - o 2
   - o 3
   - o 4
   - o 5
   - o Open text box (if more than 5 babies)

8. **Please provide your babies gestational age at birth (e.g., 36 weeks + 2 days)**
   (2 drop down windows to answer in text box for weeks and days)
   Ex what was the birth gestation in completed weeks?
   Ex what was the birth gestation in additional days?

   - o If answers for Q8 are below 37 weeks, ask Q9 and Q10

9. **Did the preterm birth of your babies affect your ability to breastfeed?**
   - o Yes
   - o No
   - o Not applicable/ I did not breastfeed

10. **Which aspects of your infants being preterm affected breastfeeding?**
    - o Underdeveloped sucking reflex
    - o Lack of energy in the babies
    - o Prolonged separation from me due to neonatal nursery admission
    - o Difficulty latching onto the breast
    - o Difficulty coordinating sucking and swallowing
    - o Need for supplemental feeding methods (e.g., tube feeding, bottle feeding)
    - o None, my preterm birth did not affect breastfeeding
    - o Not applicable/I did not breastfeed

11. **What type of birth/s did you have for your multiple birth babies? (Tick all that apply)**
    - o Spontaneous vaginal birth
    - o Assisted vaginal birth (vacuum or forceps)
    - o Breech vaginal birth
    - o Non-elective or emergency caesarean birth (decision for caesarean made after labour started)
    - o Elective caesarean birth (caesarean planned before labour started)

<u>PREGNANCY QUESTIONS</u>

12. **During your pregnancy did you plan to breastfeed your multiple birth babies?**
    - o Yes
    - o No
    - o I was unsure / undecided

13. **Did you access any breastfeeding information during your multiple birth pregnancy?**
    - o Yes
    - o No

    If Yes, please provide details - Open text box (free text)

14. **Did the cost of lactation consultants and breastfeeding education sessions influence your decision to seek support for breastfeeding your babies?**
    - o Yes, significantly
    - o Yes, somewhat
    - o No, not at all
    - o Not applicable/ I did not seek support

<u>**FEEDING YOUR BABIES AFTER BIRTH**</u>

15. **Did you ever provide your breast milk or breastfeed your multiple birth babies?**
    - o Yes
    - o No

    If answer for Q15 is No, ask Q16

16. **What were your reasons for not breastfeeding/ providing your breast milk (Tick all that apply)**
    - o I did not plan to breastfeed
    - o I was too unwell to breastfeed or express my milk after the birth
    - o My baby / babies were too unwell to breastfeed
    - o My babies were unable to latch at the breast
    - o Other (free text)

17. **Did you breastfeed/provide expressed breast milk within an hour of birth of your babies?**
    - o Yes
    - o No
    - o Unsure

    If answer for Q75 is No, ask Q18

18. **If not, please describe why your babies did not receive breast milk within an hour of birth? Tick all that apply**
    - o My babies went to the nursery soon after birth
    - o My babies were not interested in feeding in the first hour
    - o I tried but was not able to express any milk
    - o I was too unwell / receiving medical care during the first hour
    - o Other (free text)

19. **Were you satisfied with the breastfeeding support you received in hospital?**
    - o Yes
    - o No

    You are welcome to provide feedback on your breastfeeding support and suggestions on how this could be improved here (text box)

<u>**FEEDING YOUR AFTER BABIES AFTER DISCHARGE HOME**</u>

20. **What were your babies fed when your babies came home? (Tick all that apply)**

- My breastmilk by breastfeeding
- My expressed breast milk
- Donated breast milk
- Commercial infant formula

21. How did you coordinate breastfeeding when your babies first came home? (Tick all that apply)

- N/A, I did not breastfeed
- Breastfed two babies at the same time
- Breastfed one baby after the other
- Breastfed babies separately, depending on each baby's demand
- Other (free text)

22. Have you had any of the following concerns about breastfeeding or providing breastmilk for your babies? Tick all that apply

- Low milk supply
- Oversupply
- Latching difficulties
- Difficulties in positioning my babies at the breast
- Sore nipples
- Damaged nipples
- Blocked ducts
- Mastitis
- Time taken to feed
- Other (free text)

23. What lactation/breastfeeding aids have you used to assist in breastfeeding your multiple babies? Tick all that apply

- Electric breast pump
- Manual breast pump
- Nursing pillow
- Nipple shields
- Silicone milk catcher e.g., Haaka
- Supplemental nursing system
- Domperidone (Motilium) to treat low milk supply
- Galactagogues (foods/supplements to boost milk supply e.g., lactation cookies)

24. If you ever used a breast pump, what were your reasons for using one? Tick all that apply

- I have never used a breast pump
- To express milk so others could feed my babies
- To express milk for top up feeds to supplement breastfeeds
- To boost my supply
- I expressed milk to feed my babies because they did not latch at the breast
- To feed my babies when having latching issues and/or nipple pain
- To relieve engorgement
- To relieve pain and discomfort from breast conditions e.g., mastitis

25. How helpful were the following in supporting you to breastfeed in the days and weeks after birth?

	Not applicable	Very helpful	Helpful	Unsure	Unhelpful	Very unhelpful
Spouse/ partner						
Mother						
Other family members						
Friend						
Obstetrician						
Hospital midwife						
Paediatrician						
Hospital lactation consultant						
Home visiting midwife						
Child health nurse						
Lactation consultant						
Family doctor (GP)						
Australian Breastfeeding Association (helpline or in person)						
Social media (e.g., Instagram, Facebook etc.)						

26. **What aspects of care and help did you find most useful in helping you to breastfeed your multiple birth babies? Answer in Text box**

27. **What can be improved to help women breastfeed their multiple birth babies? Answer in Text box**

<u>CURRENT FEEDING</u>

28. **Are you currently breastfeeding and/or providing your breast milk for your babies?**
    - o Yes
    - o No

    If answer is No ask Q29, if answer is Yes ask Q30

29. **If no, how old were your babies when you stopped breastfeeding/ providing breast milk? Answer in Text box**

__________Month/s old

- o N/A still breastfeeding/ breast milk feeding

30. **How many times per day do you usually breastfeed each baby? (Drop down window to select amount from 0-14 for each baby)**
    Ex baby 1 = select 7, baby 2 = select 9, baby 3 = select 6

31. **How would you compare your babies breastfeeding patterns? Tick all that apply**
    - o They are the same
    - o One baby feeds more frequently
    - o One baby sucks more strongly
    - o One baby feeds more efficiently
    - o N/A my babies do not directly breastfeed

32. **How often do you usually express your milk?**
    - o Never
    - o Few times per week
    - o Once a day
    - o 2-3 times a day
    - o 4-5 times a day
    - o 6 or more times a day

33. **Select the types of foods <u>other than breast milk</u> you currently feed your babies and the average amount fed over a 24h period? (When selected provide drop down window to enter amount of times/day fed)**
    - o Expressed breast milk
    - o Commercial infant formula
    - o Cow's milk
    - o Other milk: soy milk, rice milk, goat milk, etc.
    - o Solid foods

34. **At what age (months) did you first introduce foods besides breast milk?**
    - o Not applicable, babies have only ever received breast milk
    - o Solid Food Age _________months
    - o Formula Age _________months

35. **While breastfeeding, have you ever used a feeding and sleeping schedule?**
    - o Yes
    - o No

36. **How do you manage night-time feedings for your multiple babies? Tick all that apply**
    - o My babies/ children do not wake up for night feeds anymore
    - o Feed both/all babies when one wakes up for feeding
    - o Set time for feeding during the night
    - o Feed each baby as they wake up for feeding

37. **In the first 12 months after birth which self-care activities did you participate in? Tick all that apply**
    - o Eating balanced diet

- Staying hydrated
- Light exercise
- Stress management techniques (e.g., breathing exercise, meditation….)
- Taking breaks
- Connecting with others
- Engaging in activities you enjoy
- None
- Other (free text)

Thank you for participating in this study. Results of the study will be advertised on the Centre for Human Lactation and Research Translation Facebook page after November 2024. You can follow the page here (link)
